# Surgery for synchronous and metachronous single-organ metastasis of pancreatic cancer: a SEER database analysis and systematic literature review

**DOI:** 10.1038/s41598-020-61487-0

**Published:** 2020-03-10

**Authors:** Qiaofei Liu, Ronghua Zhang, Christoph W. Michalski, Bing Liu, Quan Liao, Jorg Kleeff

**Affiliations:** 1 Department of General Surgery, Peking Union Medical College Hospital, Peking Union Medical College & Chinese Academy of Medical Sciences, Beijing, 100730 China; 2 Department of Visceral, Vascular and Endocrine Surgery, Martin-Luther-University Halle-Wittenberg, 06120 Halle (Saale), Germany; 3 Department of Surgery, Heidelberg University Hospital, Im Neuenheimer Feld 110, 69120 Heidelberg, Germany

**Keywords:** Surgical oncology, Pancreas

## Abstract

Surgery for metastatic pancreatic cancer remains controversial as the survival benefit is questionable. The aim of the present study was to analyze the survival of these patients using data extracted from the surveillance, epidemiology, and end results (SEER) program database. Further, studies on resection for metastatic disease to the lung were systematically reviewed. A total of 11,541 cases with synchronous distant metastasis were analyzed. The median survival of single-organ metastasis was better than of multi-organ metastasis (single-organ 4.0 ± 0.07 months, two-organs 3.0 ± 0.13 months, three/four-organs 2.0 ± 0.19 months; p < 0.0001). Single organ lung metastasis had longer median survival times compared to the other sites (lung 6.0 ± 0.32 months, HR 0.87, 95% CI 0.78–0.97; p = 0.013). Resection of the primary tumor was associated with longer survival in synchronous single-organ metastasis to the lung compared to no resection (14.0 ± 1.93 months vs 6.0 ± 0.31 months, p < 0.0001). A systematic literature review identified 79 cases of metachronous lung metastasis with a survival of 120.0 ± 6.32 months and 83.0 ± 24.84 months following resection of the primary tumor and metastasis, respectively. Lower TNM staging, longer interval to metastasis, and single metastatic lesion correlated with better survival. Resection in highly selected pancreatic cancer patients with synchronous and metachronous lung only metastasis might confer a survival benefit and should be considered on an individual basis.

## Introduction

Pancreatic ductal adenocarcinoma (PDAC) is one of the most aggressive malignant tumors with a rising incidence in both economically developed and developing countries^1^. It is the fourth leading cause of cancer related deaths in the US, and it is estimated to be the second leading cause in the 2030s^2^. The majority of the PDAC patients is diagnosed at an advanced stage, and only about 20% of the patients are potential candidates for oncological resection^3–5^. Most of the patients die from peritoneal spread or distant metastasis. In consideration of the relevant risk of postoperative complications and the poor survival, it has generally been accepted that for PDAC patients with metastatic disease, resection of the primary tumor or metastatic lesions is contraindicated. However, with the rapid development of new surgical techniques, more precise and minimally invasive operations, and better perioperative care, morbidity and mortality of pancreatic surgery has decreased in experienced centers^3,6^. New chemotherapeutic regimens also significantly prolong the survival of PDAC patients with metastatic tumors^7,8^. Thus, especially in the context of multimodal therapies, surgery for PDAC patients with metastasis, especially those with oligometastatic and metachronous disease, has gained interest^9,10^. Several studies have reported that for selected cases with distant metastasis, resection might relevantly prolong survival, compared to palliative treatment^9–11^.

In this study, we utilized the surveillance, epidemiology, and end results (SEER) program database and relevant reported clinical studies and case series to comprehensively generate evidence of the impact of surgery for PDAC patients with distant metastasis.

## Results

### Single-organ metastasis versus multi-organ metastasis

**Figure 1:**
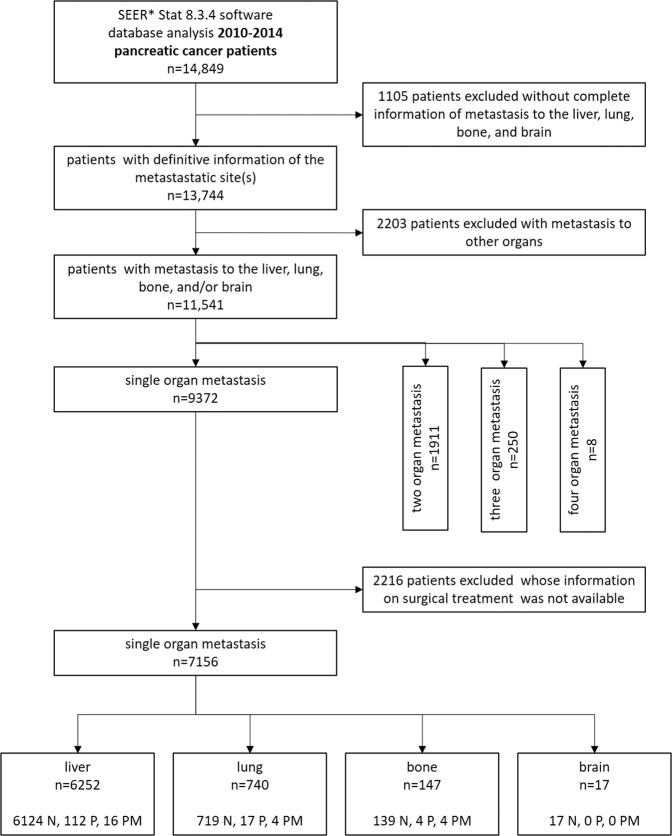
SEER database mining strategy. N: not operated; P: resection of the primary tumor; PM: resection of the primary tumor and metastasis.

A total of 11,541 patients with synchronous distant metastasis with definitive information of the site of metastasis in the liver, lung, bone and/or brain were included. A total of 9372 cases (81.2%) had single organ metastasis, 1911 cases (16.6%) had two organ metastasis, 250 cases (2.2%) had metastasis in three organs and 8 cases had metastasis in all four analyzed organs. Of the 9372 cases of single-organ metastasis, 8222 cases (87.7%) were liver, 941 cases lung (10.1%), 191 cases (2.0%) bone, and 18 cases (0.15%) brain metastasis. In 7156 out of the 9372 cases of synchronous single-organ metastasis, information was available whether the patients were operated/resected or not (Fig. 1).

The median disease specific survival (DSS) of the 11,541 patients was 4.0 ± 0.07 months (95% CI, 3.87–4.14). The 1-year, 2-year, and 3-year survival rates were 13.9%, 2.6% and 0.6%, respectively (Fig. S1A, Table S1). After multivariate analysis, tumors in the body or tail of the pancreas (Fig. S1B), older age (Fig. S1C), multi-organ metastasis (Fig. S1D), and poorly/undifferentiated tumors (Fig. S1E) were independent risk factors for worse prognosis (Table S1). The gender of the patients and T staging of the primary tumor did not correlate with DSS (Table S1 and Fig. S2).

### Single-organ lung metastasis versus other sites of single-organ metastasis

**Figure 2:**
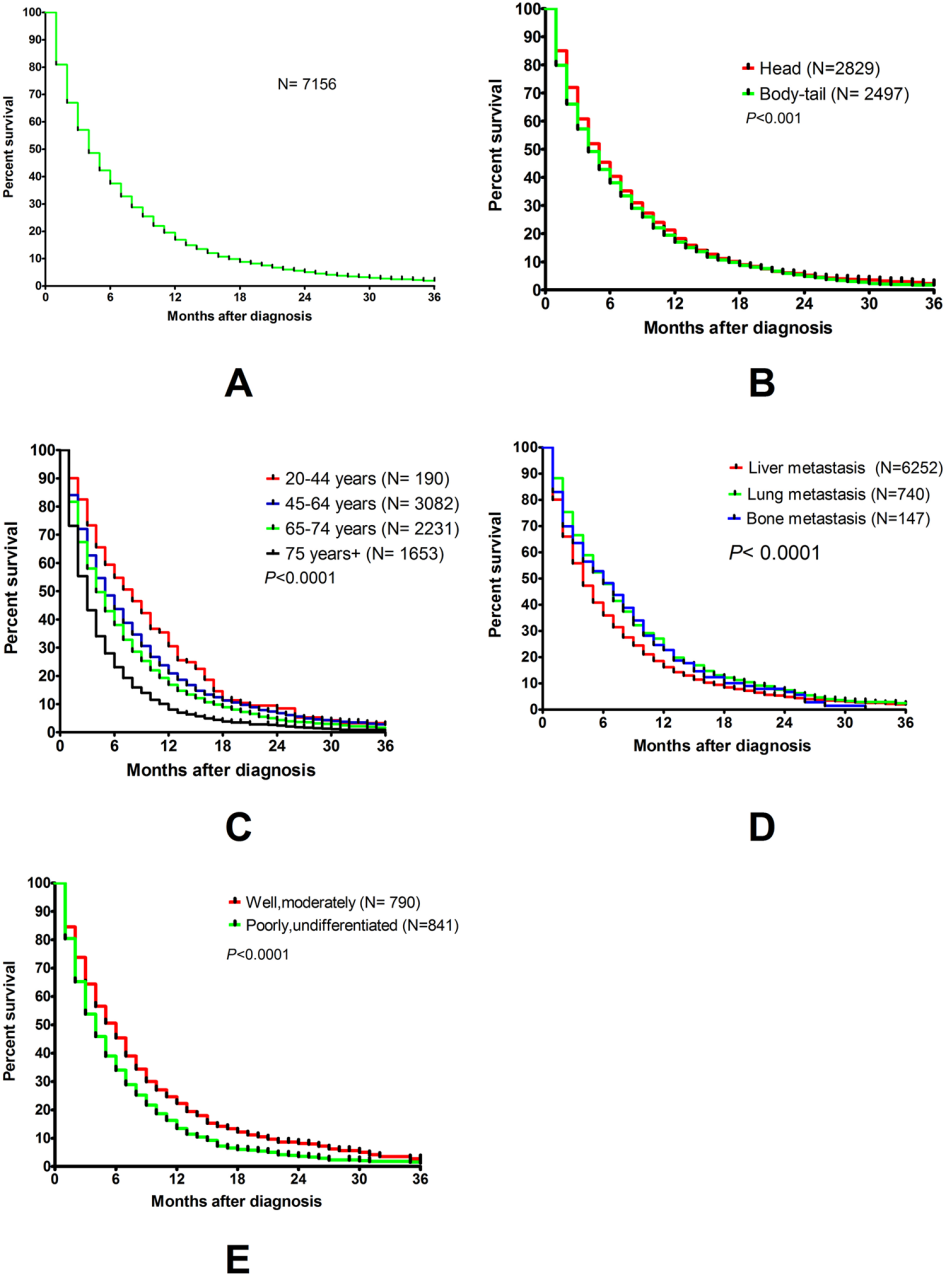
Survival curves for 7156 cases of PDAC patients with single organ metastasis (**A**), for different locations of the tumor (**B**, p < 0.05), for different age groups (**C**, p < 0.001), for different involved organs (**D**, p < 0.001), and for different grades of differentiation (**E**, p < 0.001).

A total of 7156 cases of synchronous single-organ metastasis with definitive surgical information were included. The 1-year, 2-year, and 3-year overall survival rates were 16.9%, 5.1%, and 1.9%, respectively. Patients with single-organ metastasis between 20–44 years of age had a relative better prognosis with a median survival of 8.0 ± 0.88 months as compared to other age groups (Table 1). Tumors located in the pancreatic head had a slightly better prognosis than those in the body or tail of the pancreas (5 ± 0.14 months vs 4 ± 0.14 months; *p* = 0.015). Well or moderate differentiation predicted better survival compared to poorly/undifferentiated tumors (6 ± 0.31months vs 4 ± 0.20 months; *p* < 0.0001). Lung metastasis had better survival than other metastasis (lung metastasis 6.0 ± 0.32 months, bone metastasis 6.0 ± 0.99 months, liver metastasis 4.0 ± 0.86 months, brain metastasis 4.0 ± 0.68 months; lung versus other metastatic sites HR: 0.87 95% CI: 0.78–0.97; p = 0.013) (Table 1). Lung metastasis had the best 1-year overall survival rate (19.5%), compared to liver metastasis (13.9%), bone metastasis (17.0%) and brain metastasis (0%). The median DSS of these single-organ metastatic patients was 4.0 ± 0.08 months (Fig. 2A). Tumor location in the body and tail (Fig. 2B), older age (Fig. 2C), metastatic site (Fig. 2D) and poorly/undifferentiated tumors (Fig. 2E) correlated with survival. However, after multivariate analysis, only age, differentiation, and metastatic site were independent risk factors for survival (Table 1). The gender of the patients and T staging of the primary tumor did not correlate with survival (Table 1 and Fig. S3).

**Table 1:** Univariate and multivariate analysis of factors influencing DSS in 7156 PDAC patients with single-organ metastasis.

Variable		N	1-year survival rate	2-year survival rate	3-year survival rate	Median survival ± SE (M)	Univariate analysis		Multivariate analysis		
							95% CI	*p* value	HR	95% CI	*p* value
Single-organ metastasis		7156	16.95%	5.09%	1.95%	4.0 ± 0.08	3.84–4.16				
Gender	female	3296	16.74%	5.28%	1.82%	4.0 ± 0.12	3.77–4.23	>0.05			
	male	3860	17.11%	4.93%	2.00%	4.0 ± 0.11	3.78–4.22				
Age	20–44 years	190	30.51%	8.49%	NA	8.0 ± 0.88	6.28–9.17	<0.0001 (20–64 years/65+ years)	1:1.24 (20–64 years/65+ years)	1.16–1.36	<0.0001
	45–64 years	3082	20.90%	6.82%	2.65%	5.0 ± 0.15	4.72–5.28				
	65–74 years	2231	16.84%	4.36%	NA	4.0 ± 0.14	3.72–4.28				
	75+ years	1653	8.09%	2.43%	NA	3.0 ± 0.10	2.80–3.20				
Localization	head	2829	21.26%	5.24%	2.27%	5.0 ± 0.14	4.72–5.28	0.015 (head/body and tail)			>0.05
	body-tail	2496	19.44%	4.80%	1.73%	4.0 ± 0.14	3.72–4.28				
	unknown	1831	NA	NA	NA	NA					
Differentiation	G1, G2	790	22.27%	8.15%	1.82%	6.0 ± 0.31	5.39–6.61	<0.0001 (G1-2/G3-4)	1:1.31 (G1-2/G3-4)	1.17–1.50	<0.0001
	G3, G4	841	13.41%	3.54%	NA	4.0 ± 0.20	3.61–4.39				
	unknown	5524	NA	NA	NA	NA					
T classification	T4	1376	17.35%	4.59%	NA	5.0 ± 0.11	4.79–5.21	>0.05			
	T1-T3	4139	18.73%	5.85%	2.10%	5.0 ± 0.20	4.60–5.40				
	unknown	1641	NA	NA	NA	NA					
Metastatic site	liver	6252	13.87%	2.99%	0.6%	4.0 ± 0.09	3.83–4.17	<0.0001 (others/lung)	1:0.87 (others/lung)	0.78–0.97	0.013
	lung	740	19.46%	4.60%	0.67%	6.0 ± 0.32	5.37–6.63				
	bone	147	17.00%	NA	NA	6.0 ± 0.99	4.05–7.94				
	brain	17	0	0	0	4.0 ± 0.08	2.67–5.33				

NA: not available.

### Resection of the primary tumor and survival for single-organ liver and lung metastasis

**Figure 3:**
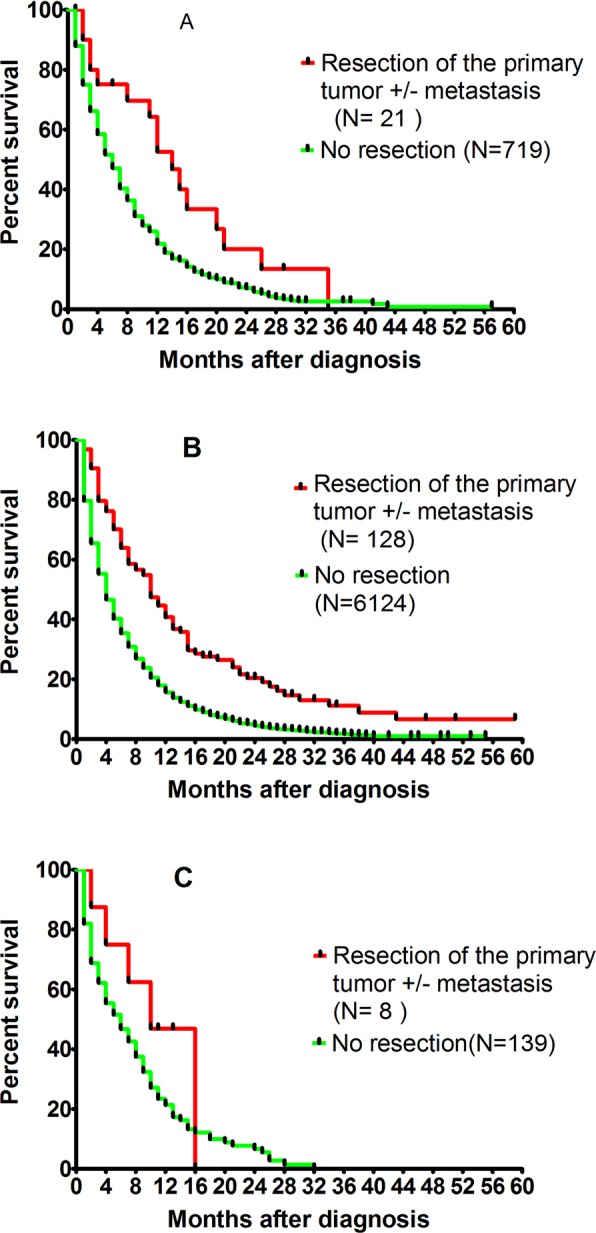
Survival curves of single organ metastasis depending on the resection of the primary tumor with or without resection of metastasis, for lung metastasis (**A**, p < 0.001), liver metastasis (**B**, p < 0.001), and bone metastasis (**C**, p > 0.05).

Among 7156 patients, a minor proportion underwent resection. 128 cases of liver metastasis, 21 cases of lung metastasis, 8 cases of bone metastasis and no case of single-organ brain metastasis underwent resection for the primary tumor with or without resection of metastasis. When compared to the non-operated cases, operated cases of liver metastasis and lung metastasis had a relative longer survival following resection of the primary tumor: lung metastasis, 14.0 ± 1.93 months vs 6.0 ± 0.32 months, *p* < 0.0001; liver metastasis, 10.0 ± 1.16 months vs 4.0 ± 0.09 months, *p* < 0.0001) (Fig. 3).

16 out of 128 cases with liver metastasis, and 4 out of 21 cases with lung metastasis underwent simultaneous resection of the primary tumor and metastasis. Although there was no significant difference, there was a trend for better survival in the group of simultaneous resections of the primary tumor and metastasis compared to the group that underwent resection of the primary tumor only for both lung and liver metastasis (Fig. S4).

### Systematic review of metastasis resection for metachronous single-organ lung metastasis

Twenty studies, including 401 patients, which reported the outcomes of PDAC patients with synchronous or metachronous single-organ lung metastasis were collected (Fig. S5). All published data were cases reports or case series, there was no prospective clinical trial.

Four scenarios of single-organ lung metastasis were initially analyzed (Table S2). (i): synchronous metastasis with non-surgical treatment (1 study). Kruger *et al*.^12^ reported 13 cases with a median survival of 22.8 months (range 17.3–28.3 months). (ii): metachronous metastasis from unresectable PDAC, with non-surgical treatment (1 study). Kruger *et al*.^12^ reported 5 cases with a median survival after diagnosis of metastasis of 10.7 months (rang, 0–24.9 months). (iii): metachronous metastasis after radical resection of the primary tumor, with non-surgical treatment of the metastasis (8 studies); These 8 studies reported on 212 cases. The median survival after initial resection and diagnosis of lung metastasis ranged from 23.0–50.5 months and 7.5–31.3 months, respectively. (iv): metachronous metastasis after resection of the primary tumor, with surgical treatment of the metastasis (17 studies). These 17 studies (including 154 cases) reported a median survival after the initial resection and resection of metastasis of 19.0–92.0 months and 16.0–37.0 months, respectively.

**Figure 4:**
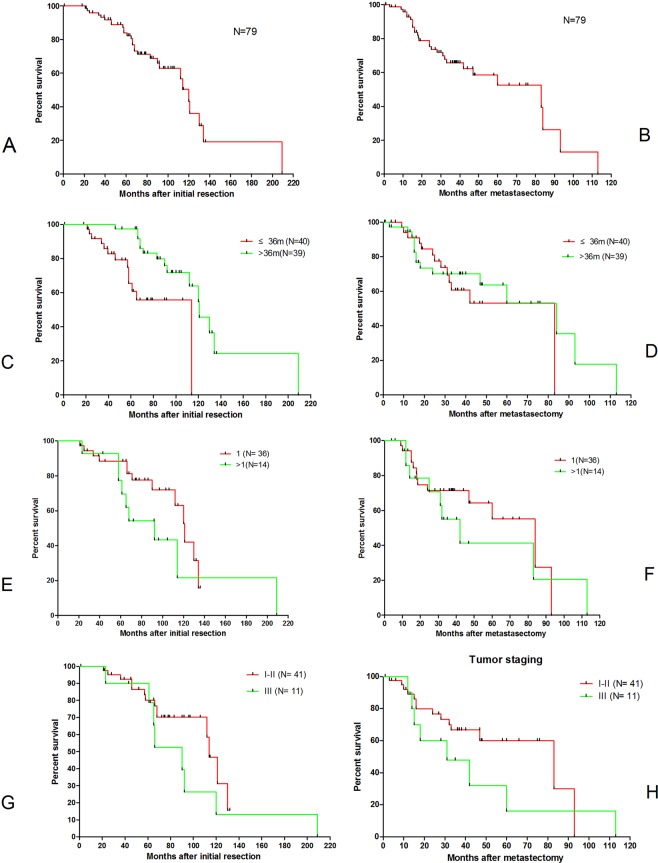
Survival curves of 79 cases of metachronous single organ lung metastasis undergoing resection of metastasis: after the initial operation (**A**), after resection of metastasis (**B**). Survival depending on different intervals to metastasis: after the initial operation (**C**, p < 0.05), after resection of metastasis (**D**, p > 0.05). Survival depending on different number of metastatic lesions: after the initial operation (**E**, p > 0.05), after resection of metastasis (**F**, p > 0.05), Survival depending on TNM staging: after the initial operation (**G**, p > 0.05), after resection of metastasis (**H**, p > 0.05).

Of those 154 cases from 17 studies, only 79 cases from 11 studies were available with complete survival information for each case and were extracted for further quantitative synthesis (Tables 2 and S3). Most of these patients underwent adjuvant chemotherapy after resection of metastasis. No surgical mortality was reported. The median time to recurrence after the initial resection was 36 months, ranging from 3 to 96 months. After quantitative synthesis of these 79 cases, the median survival after the initial resection and resection of metastasis was 120.0 ± 6.32 months and 83.0 ± 24.84 months, respectively (Fig. 4A,B). A longer interval (>36 months) was an indicator for better prognosis after the initial resection but not following resection of metastasis (Fig. 4C,D). Single metastatic lesions (Fig. 4E,F) and lower category of initial TNM staging (Fig. 4G,H) were associated with non-significant increase in survival following the initial resection as well as following resection of metastasis. Age and gender did not correlate with the survival after the initial resection or resection of metastasis (Fig. S6).

**Table 2:** Summary of the literature review of 79 PDAC patients with single organ metachronous pulmonary metastasis undergoing resection of the primary tumor as well as resection of metastasis.

	Number of patients
**Age**	
<65	18
≥65	35
NK	26
**Gender**	
male	20
female	23
NK	36
**Initial TNM staging(7th)**	
I	9
II	33
III	10
NK	27
**Number of metastatic lesions**	
1	36
>1	14
NK	29
**Adjuvant treatment after resection**	
yes	69
no	3
NK	7
**Living status**	
alive	51
dead	28
**Mortality**	
No	79
Yes	0

NK: not known.

## Discussion

Radical resection of both primary and metastatic lesions has been carried for an increasing number of tumor types with promising results^13,14^. For pancreatic cancer with metastasis, resection of either the primary tumor or metastasis is generally not indicated; however, for cases with limited (oligo-) metastatic lesions, which could potentially be resected, surgery has been advocated on an individual basis in some experienced pancreatic cancer centers^9,15^.

One reason being that during the last years, more effective chemotherapeutic regimens, such as Folfirinox or gemcitabine plus nab-paclitaxel, have significantly prolonged the survival of metastatic pancreatic cancer patients. In case of initially unresectable cases or limited metastatic spread, these effective therapies have given some patients the chance for resection of the primary tumor or resection of both primary tumor and metastasis^11,16–18^. Obviously, the most important point is to select those patients that might benefit from this aggressive surgical approach^17^.

Although the liver is the most common site of distant metastasis of pancreatic cancer, the lung ranks second. Single-organ metastasis to the lung are not common, but several studies of small cohorts have reported a better prognosis than for other types of metastasis, including distant lymph node metastasis, liver metastasis, and peritoneal metastasis, which indicates that the metastatic site is another important aspect which should be taken into account when considering surgical treatment for metastatic pancreatic cancer patients^19–21^.

According to our SEER database analysis, synchronous multi-organ metastasis had worse survival compared to single-organ metastasis, and synchronous single-organ metastasis to the lung had better survival compared to other metastatic sites. This is in line with a recent analysis that also demonstrated better survival for isolated lung metastasis compared to isolated liver metastasis^22^. We have also shown that for synchronous single-organ metastasis, older age, worse differentiation, and non-resection of the primary tumor were independent risk factors for worse survival and that survival was better for resected versus non resected synchronous single-organ metastasis to the lung and liver.

Many factors, such as the general physical status, tumor burden, any additive/palliative chemotherapy/radiotherapy among others, could affect patient survival. Information regarding these items are either absent or incomplete in the SEER database. Importantly, information regarding chemo-/radiotherapy is insufficient and it is therefore officially suggested not to analyze its impact on survival. Together, the extent of selection and treatment bias is difficult to estimate. Obviously, only few patients with synchronous single-organ metastasis to the lung or the liver would benefit from resection. The present study provides some information on how to select potential candidates for resection. For both multi-organ and single-organ metastasis, younger age and better tumor differentiation were independent factors for increased survival in the SEER database analysis.

A systematic review of the available clinical reports on resection for metachronous single-organ lung metastasis revealed a striking median survival of 83.0 months (95% CI 34.31–131.69 months) after resection of metastasis. Single metastatic lesions, initial lower TNM staging, and longer interval from primary resection to recurrence were associated with increased survival, although this was not statistically significant.

Since resection of pulmonary metastasis can be carried out safely with low morbidity and mortality rates, resection could be considered for those patients with a longer interval between resection of the primary tumor and detection of lung-only metastasis, better differentiated tumor, younger age, and single metastatic lesions.

It has been reported that the occurrence of pulmonary metastasis is generally later than that of liver metastasis, and it has also been proposed that the biological behavior of PDAC presenting with a first site of metastasis other than the liver may be related to its molecular phenotype^23^. Thus, pancreatic cancer with Her-2 amplifications were noted to have less frequent liver metastases and an increased frequency of lung and brain metastasis^24^. SMAD4 loss was more common in patients with widespread metastatic disease than locally advanced tumors^25^. Molecular analysis has helped to identify those patients that may benefit from resection of metastasis of colorectal cancer^25^. In the future, molecular subtyping might also help to identify key signaling pathways and potential surrogates to select metastatic pancreatic cancer patients for whom surgical resection might be beneficial^26–28^. Since there are no prospective, randomized trials, solid evidence of criteria for patient selection is lacking.

Although this is a comprehensive retrospective study based on the SEER database and a systematic analysis of published reports, there are limitations. (1) Data of systemic treatment of the patients included in the SEER database is incomplete. Therefore, important prognostic factors could not be analyzing in this context. (2) There is a substantial selection bias regarding patients that were resected and those that were not resected in the SEER database. (3) There are no randomized clinical trials and all the published reports were either case series or case reports, and (4) published cases were highly selected, resulting in a significant selection bias.

In conclusion, most pancreatic cancer patients with distant metastasis have a poor prognosis. In contrast to other metastatic sites, resection of synchronous and metachronous single-organ lung metastasis shows promising results in terms of safety and survival in highly selected patients. Younger age, better tumor differentiation, longer interval between the initial resection and diagnosis of metastasis, and single metastatic lesions predict better outcome, and all patients should be treated with chemotherapy first. Patient selection is important as expansion of this practice may offer little advantage or even harm patients.

Further, it is currently not clear whether there is a true benefit for resection, or whether the data merely reflect a selection bias of patients with a better prognosis independent of resection. Clinical trials or at least multi-center, prospective data are needed to evaluate the outcome of surgical treatment for both synchronous and metachronous single-organ lung metastasis in pancreatic cancer patients.

## Methods

### Mining of the SEER database

The clinicopathological, surgical and survival information of PDAC patients with distant metastasis were extracted from the SEER database (2010–2014) by using the SEER* Stat 8.3.4 software. The inclusion criteria were as follows: (1) Patients had to be 20 years or older, be pathologically diagnosed with PDAC from January 1, 2010 to December 31, 2014; (2) patient had active follow-up, and did not suffer any other kind of malignant tumor; (3) the information of metastasis in liver, lung, bone, and brain was available. The exclusion criteria were as follows: (1) Patient died from any other cause which was not related to PDAC; (2) metastasis at any other site, besides liver, lung, bone and brain; (3) the information of surgery for the primary tumor or metastatic lesion was missing.

The setting of mining strategies of the SEER database was as follows: “cases in research database”, “known age”,“age at diagnosis = 20–85 + ”, “year of diagnosis = 2010–2014”,“site recode ICD-3/WHO 2008 = pancreas”, “malignant behavior”, “diagnostic confirmation = microscopically confirmed”, “ICD-0-3His/behav, malignant = 8140/3 and 8500/3”. Details of the data extraction are presented in Fig. 1. To ensure follow-up of at least three years, patients were included until December 2014.

The following variables were extracted: Patient ID, age, sex, year of diagnosis, vital status recode, survival months (>0), COD to site recode, SEER cause-specific death classification, type of follow-up expected, survival months flag, CS mets DX-bone (2010+) (bone metastasis), CS mets at DX-brain (2010+) (lung metastasis), CS mets at DX-liver (2010+) (liver metastasis) and CS mets at DX-lung (2010+) (lung metastasis) and RX Summ–Surg Prim Site (1998+)(surgery for primary site), RX Summ–Scope Reg LN Sur (2003+) (surgery for lymph node) and RX Summ–Surg Oth Reg/Dis (2003+) (surgery for metastatic lesion or the others). All patients were initially diagnosed as stage IV, thus all patients had synchronous metastasis. Since the information of adjuvant treatment is largely incomplete and sometime it could be misleading, it is officially suggested not to analyze the survival impact of adjuvant treatment by using data from SEER database. Therefore, in this study, the information of adjuvant treatment was not analyzed.

### Systematic review

We comprehensively searched the published English literature for cases of PDAC with lung metastasis in the Medline database (between 1^st^, January 1990 and 1^st^, October 2019) and Science Citation Index Expanded database (between 1^st^, January 1990 and, 1^st^, October 2019). The Cochrane Central Register of Controlled Trials (CENTRAL) and ClinicalTrial.org (update to1^st^, October 2019) were also searched to identify randomized controlled trials and non-randomized studies following the Preferred Reporting Items for Systematic reviews and Meta-analysis (PRISMA) guidelines. Both non-randomized (retrospective studies, prospective studies, case reports, case series) and randomized studies were collected for further analysis. Conference abstracts were excluded.

The following searching strategy was used with the combination of “pancreatic cancer OR pancreatic adenocarcinoma” AND “lung OR pulmonary” AND “metastasis or metastatic”. The literature review was independent performed by Q.F. Liu and R.H Zhang, and the selection of the literature discussed together. Most of the papers were duplicated in different databases.1309 papers were left after removal of duplications. Titles and abstracts were reviewed, and 1006 papers were excluded because of obvious non relevance. After further careful review of the abstracts of 303 papers, only 20 papers fulfilled our criteria. Here, the treatment and survival of both synchronous and metachronous single organ lung metastasis of pancreatic cancer with or without surgical resection were recorded.

Information including country, publication year, patient age, gender, tumor differentiation, tumor stage, primary treatment, number and location of metastatic lesions, treatment for metastatic lesions, survival status, interval time between first and second operation, adjuvant treatment, overall survival time after the initial operation, survival time after the second operation, and mortality of surgery was extracted and analyzed.

### Statistical analysis

Disease specific survival (DSS) was defined as duration from the date of diagnosis to the date of death and recorded as median value ± standard error (SE) with 95% confidence intervals (CI). Survival time was defined as censored if the patients were alive at the last follow-up. The DSS were analyzed by using Kaplan-Meier curves and log-rank tests. Multivariate analysis was performed with a logistic regression model to identify factors predicting survival. All tests were two-tailed and a *p* value of less than 0.05 was considered statistically significant. Analyses were carried using GraphPad Prism 5.0 and IBM SPSS statistics 21 (SPSS Statistics V21, IBM Corporation, New York).

## Supplementary information

Supplemental Information.

## Data Availability

Primary data is available upon request.
