# Supplementary material for: Surgery for synchronous and metachronous single-organ metastasis of pancreatic cancer: a SEER database analysis and systematic literature review
Source: Sci Rep. 2020 Mar 10;10:4444. doi: 10.1038/s41598-020-61487-0 (PMC7064579; doi:10.1038/s41598-020-61487-0)
Supplement: Supplementary file 1 — Supplemental Information. [file 41598_2020_61487_MOESM1_ESM.pdf]

Supplementary material

Surgery for synchronous and metachronous single-organ metastasis of pancreatic cancer: a SEER database analysis and systematic literature review

Qiaofei Liu[1], Ronghua Zhang[1],Christoph W. Michalski[2], Bing Liu[3], Quan Liao[1], Jorg Kleeff[2]

1. Department of General Surgery, Peking Union Medical College Hospital, Peking Union Medical College & Chinese Academy of Medical Sciences, Beijing 100730, China

2. Department of Visceral, Vascular and Endocrine Surgery, Martin-Luther-University Halle-Wittenberg, 06120 Halle (Saale), Germany

3. Department of Surgery, Heidelberg University Hospital, Im Neuenheimer Feld 110, 69120 Heidelberg, Germany

Address for correspondence:

Jorg Kleeff, MD, FACS, FRCS, Department of Visceral, Vascular and Endocrine Surgery, University Hospital Halle (Saale), Martin-Luther-University Halle-Wittenberg

Ernst-Grube-Str. 40, 06120 Halle (Saale), Germany

phone: +49-345-557-2314, fax: +49-345-557-2551

email: joerg.kleeff@uk-halle.de

Quan Liao, MD, Department of General Surgery, Peking Union Medical College Hospital, Peking Union Medical College & Chinese Academy of Medical Sciences,

1# Shuai Fu Yuan, Dong Chen District, 100730 Beijing, China

phone: +86-10-6915-6007

email: lqpumc@126.com

Suppl. figures and tables

Figure S1: Survival curves for the whole cohort of 11541 PDAC patients with distant metastasis (A), depending on different locations of the tumor (B, p<0.05), depending on different age groups (C, p<0.001), depending on the number of involved organs (D, p<0.001), and depending on different grades of differentiation (E, p<0.001).

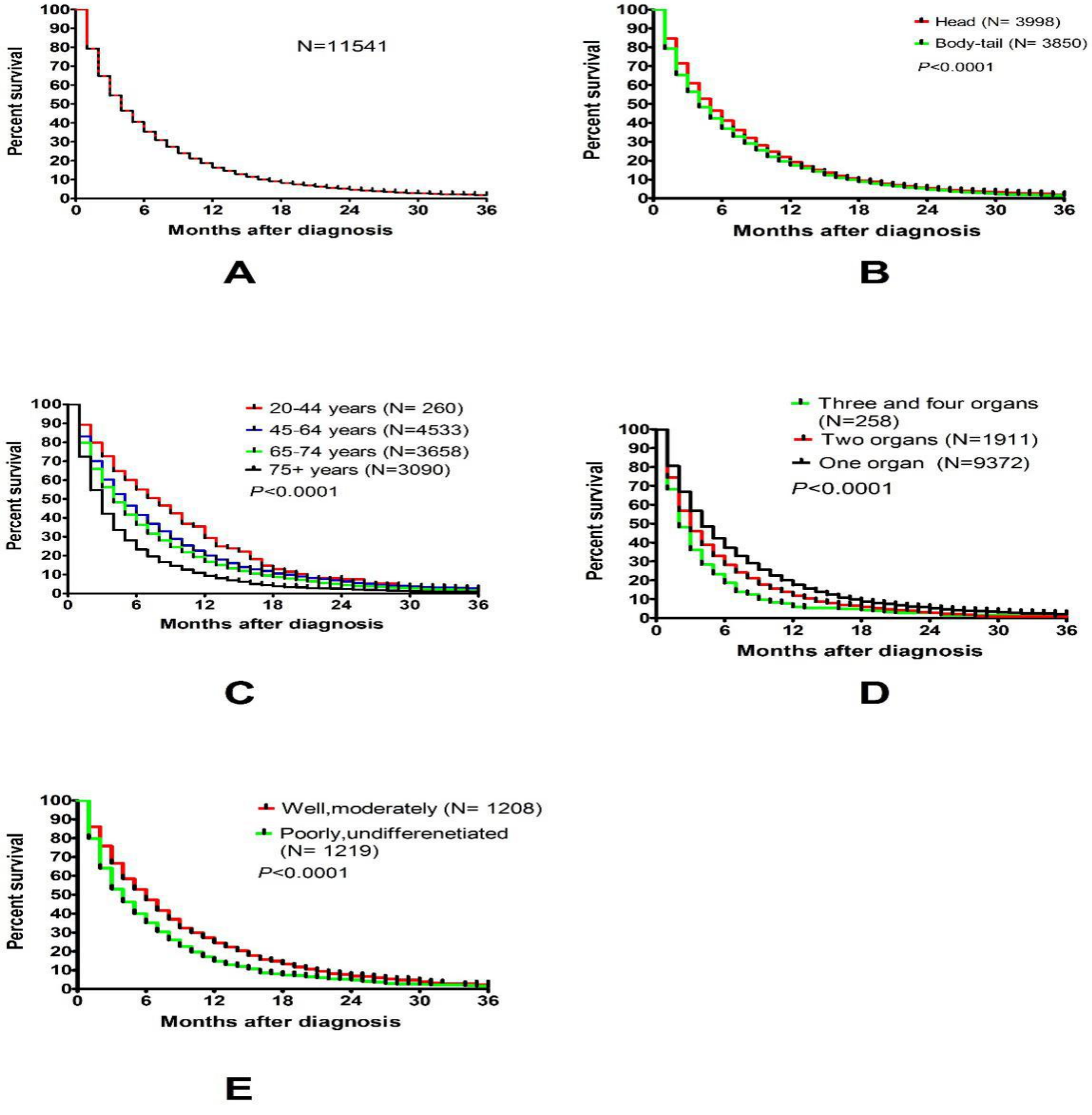

Figure S2: Survival curves for the whole cohort of 11541 PDAC patients with distant metastasis, depending on T stage (A, p>0.05), depending on gender (B, p>0.05).

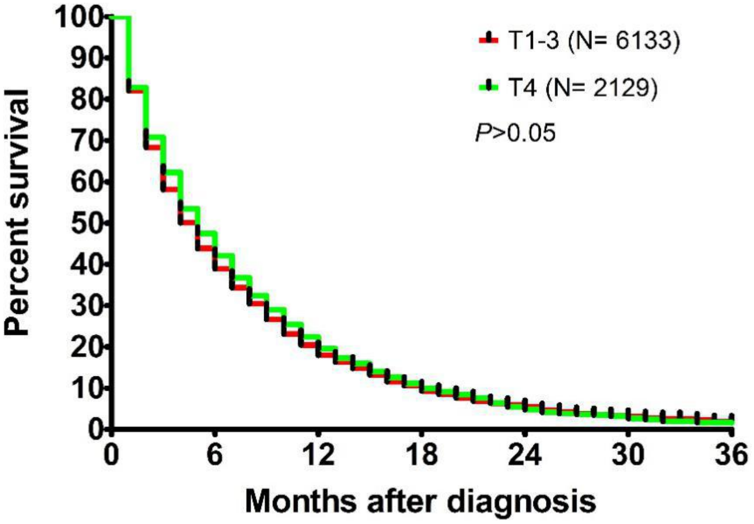

A

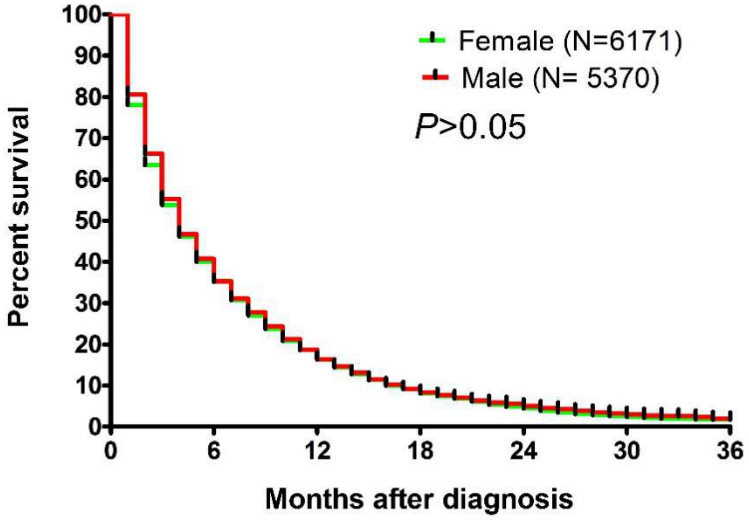

B

Figure S3: Survival curves for the whole cohort of 7156 PDAC patients with single organ distant metastasis, depending on gender (A, p>0.05), depending on T stage (B, p>0.05).

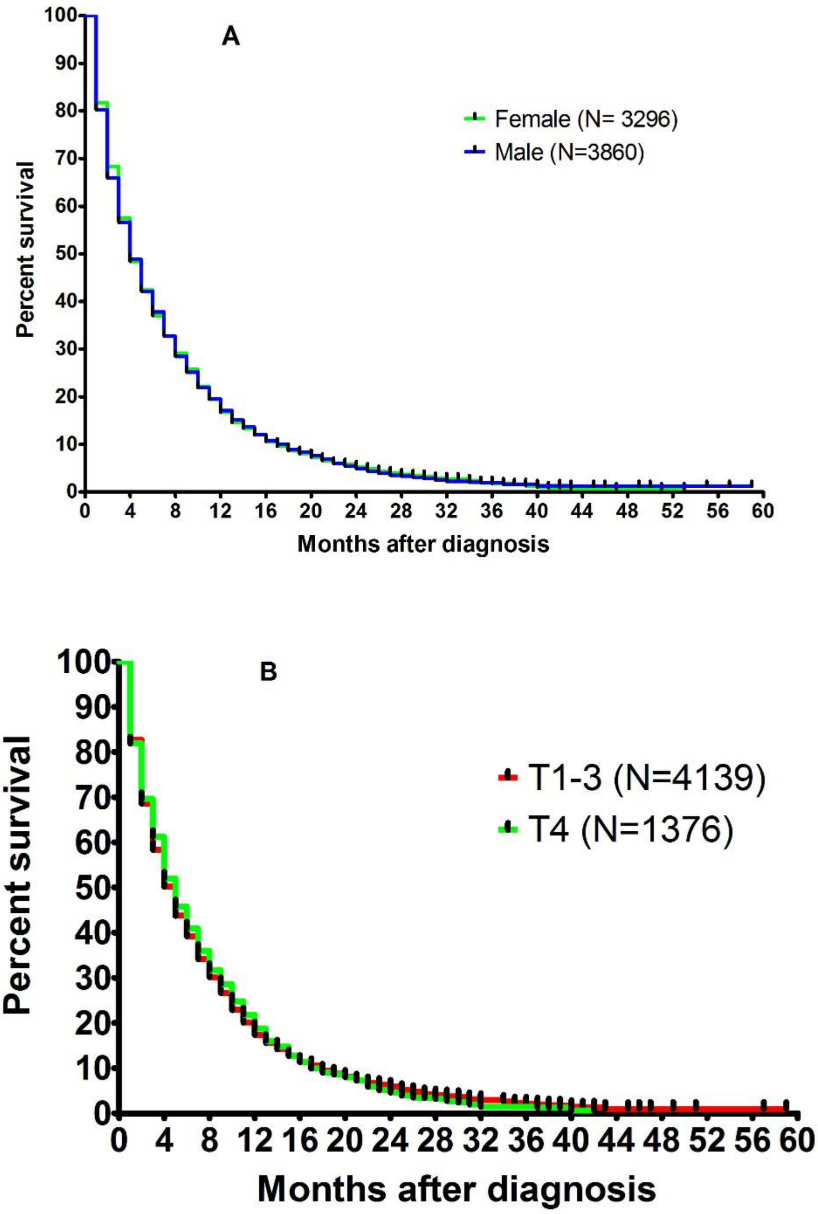

Figure S4: Survival curves of single organ liver metastasis undergoing primary tumor resection with or without resection of metastasis (A, p>0.05) and single organ lung metastasis undergoing primary tumor resection with or without resection of metastasis (B, p>0.05)

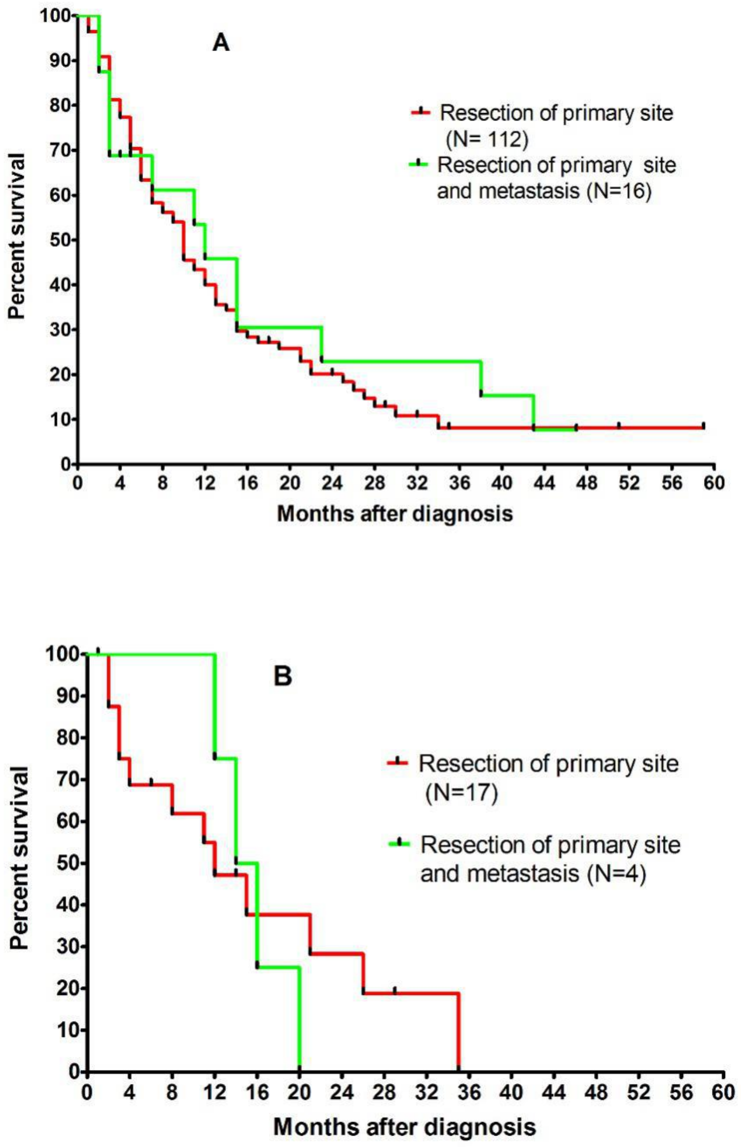

Figure S5: PRISMA flowchart of the literature review

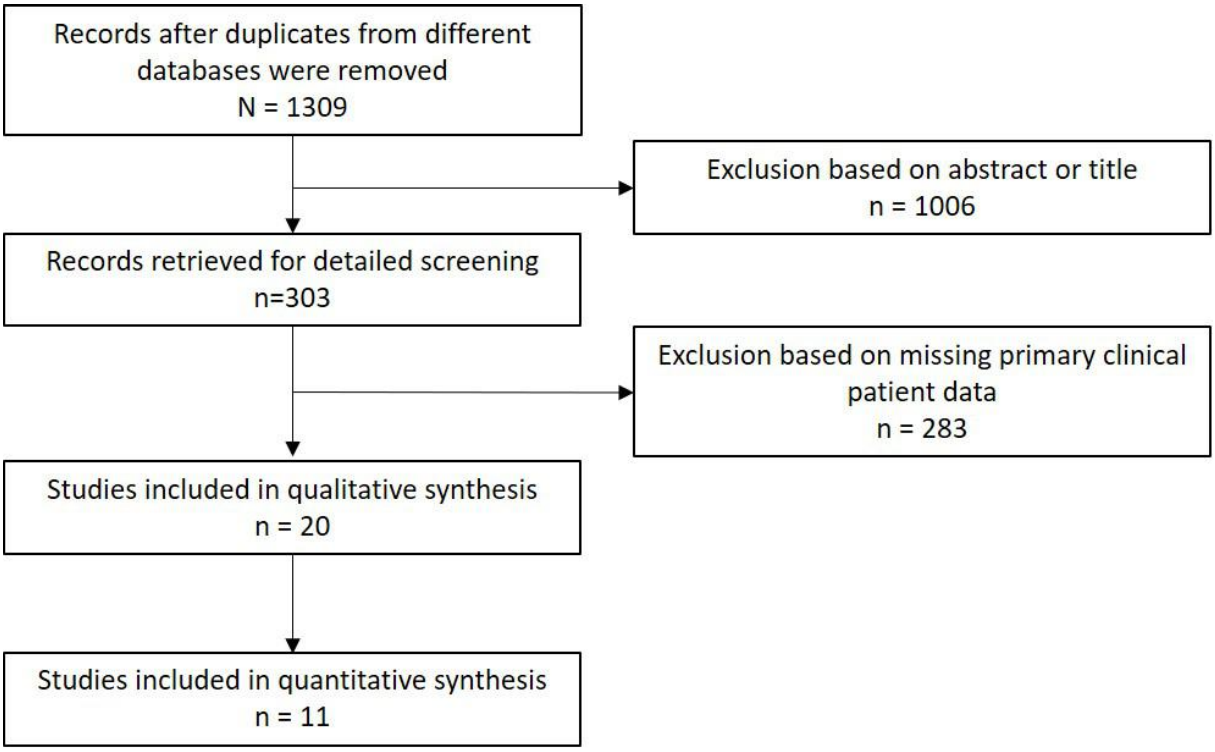

Figure S6: Survival curves of PDAC patients with metachronous single organ lung metastasis depending on age (survival from initial resection A, from resection of metastasis B), gender (survival from initial resection C, from resection of metastasis D).

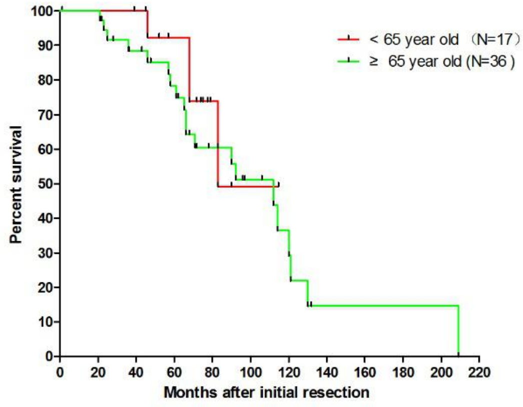

A

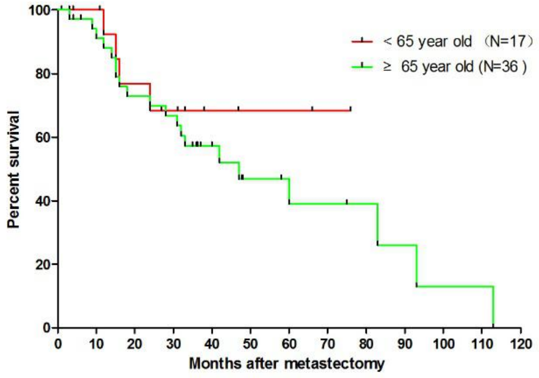

B

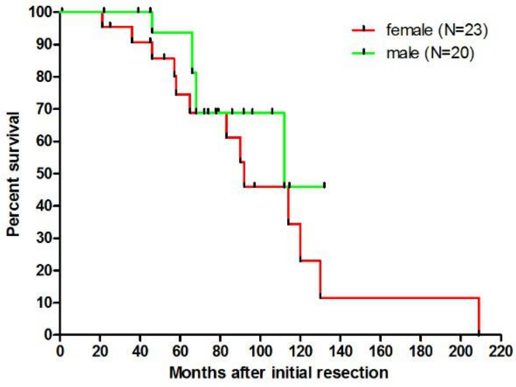

C

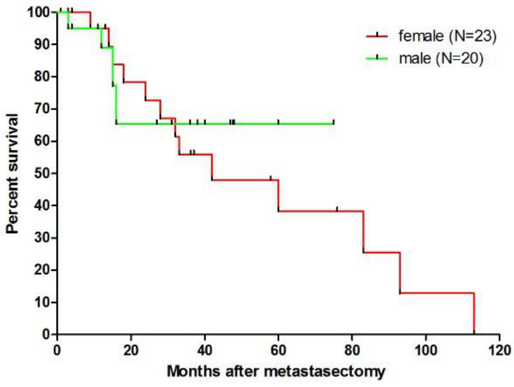

D

Table S1: Univariate and multivariate analysis of factors influencing DSS in 11,541 PDAC patients with distant metastasis.

Variable		N	1- year survival rate	2- year survival rate	3- year survival rate	Median survival ±SE(m)	Univariate analysis		Multivariate analysis		
							95% CI	*p* value	HR	95% CI	*p* value
Whole cohort		11541	13.89%	2.62%	0.62%	4.0±0.07	3.87-4.14				
Gender	female	6171	11.76%	2.79%	0.42%	4.0±0.10	3.381-4.19	>0.05			
	male	5370	11.75%	3.11%	0.59%	4.0± 0.97	3.81-4.19				
Age	20-44 years	260	27.30%	4.61%	NA	8.0±0.73	6.57-9.44	<0.0001 (20-64 years/ 65+ years)	1:1.22 (20-64 years/ 65+ years)	1.19-1.25	<0.0001
	45-64 years	4533	16.74%	3.42%	0.70%	5.0±0.12	4.76-5.24				
	65-74 years	3658	14.02%	2.57%	0.49%	4.0±0.12	3.77-4.23				
	75+ years	3090	8.31%	1.33%	0.16%	3.0±0.06	2.89-4.11				
Localization	head	3998	16.83%	3.15%	0.65%	5.0±0.12	4.76-5.24	<0.0001 (head/ body and tail)	1:1.11 (head/ body and tail)	1.00-1.26	0.04
	body/tail	3851	14.62%	2.68%	0.36%	4.0±0.12	3.78-4.23				
	unknown	3692	NA	NA	NA	NA					
Differentiation	G1, G2	1208	21.27%	4.30%	0.66%	6.0±0.27	5.48-6.52	<0.0001 (G1-2/G3-4)	1:1.35 (G1-2/G3-4)	1.22-1.49	<0.0001
	G3, G4	1219	13.29%	3.20%	0.57%	4.0±0.18	3.65-4.36				
	unknown	10114	NA	NA	NA	NA					
T classification	T4	2129	19.58%	4.87%	NA	5.0±0.91	4.82-5.18	>0.05			

	T1-T3	6133	18.00%	5.49%	1.83%	5.0±0.17	4.68-5.32				
	unknown	3279	NA	NA	NA	NA					
Number of involved organs	one organ	9372	14.94%	2.91%	0.68%	4.0± 0.07	3.86-4.14	<0.0001 (one organ/ multiple organs)	1:1.33 (one organ/ multiple organs)	1.21-1.45	<0.0001
	two organs	1911	9.79%	1.26%	0.21%	3.0±0.13	2.75-3.25				
	three/four organs	258	6.20%	1.16%	NA	2.0±0.19	1.62-2.38				

NA: not available

Table S2: Systematic literature review of single-organ lung metastasis of PDAC

Reference	Country	Year	n	Age<br>Median (range)	Gender<br>(m/f)	Interval (m)<br>Median (range)	Status	Survival 1<br>Median (range)	Survival 2<br>Median (range)	Type
Kruger et al.[1]	Germany	2016	13	NA	NA	0		22.8(17.3-28.3)		i
Kruger et al. [1]	Germany	2016	5	NA	NA	5.0(3.4-8.0)		NA	10.7(0-24.9)	ii
Arnaoutakis et al.[2]	USA	2011	22	67.4 (11.2)<br>(mean, SD)	11/11	14 (8–20)		23 (18–52)	7.5 (3.4–22.0)	ii
Yamashita et al.[3]	Japan	2015	13	NA	NA	15.3(.1-72)		37.5 (3.2-73.4)	17.1(0.4-37.5)	iii
Downs-Canner et al[4]	USA	2015	23	NA	NA	NA		33.8 (NA)	NA	iii
Wangjam et al.[5]	USA	2015	28	65.2 (47-80)	9/15<br>(4, unknown)	12.7 (9.9-30.8)		27.8 (18.2-50.0)	8.5(5.7-19.5)	iii
Kruger et al.[1]	Germany	2016	22	NA	NA	10.5 (1.7-65.7)		NA	31.3 (23.8-33.8)	iii
Zheng et al. [6]	Japan	2017	24	9/15<br>(<65, >65)	16/8	15(NA)		36(NA)	20 (NA)	iii
Lovecek et al.[7]	Japan	2017	3	NA	0/3	22 (11.5-32)		50.5 (24-82.5)	18.5 (12.5-60.5)	iii
Groot, et al.[18]	USA	2018	32	70 (63 ‑ 79)	NA	NA		24.5 (20.6 ‑ 28.5)	8.1 (4.4 ‑ 11.7)<br>(supportive	iii

									treatment)	
Groot, et al.[18]	USA	2018	45	69 (63 – 77)	NA	NA		34.2 (23.4 – 45.1)	20.2 (17.2 – 23.2) (chemo/radiation)	iii
Thomas et al. [8]	USA	2012	7	NA	NA	52 (14–86)		63.7 (18-100.3) (DFI)	19 (0.7–48) (DFI)	iv
Miyasaka et al. [9]	Japan	2018	1	70	male	56;58;74	alive	96	40	iv
Yasukawa et al.[10]	Japan	2017	12	74.25 (63-83)	6/6	NA		66.5 (23-121)	32 (6-66)	iv
Lovecek et al. [7]	Japan	2017	2	NA	0/2	34/52	alive/alive	45/65	11/13	iv
Decoster et al.[11]	France	2016	37	70(54-86)	10/27			31.9 (NA)	20.8 (NA)	iv
Nakajima et al. [12]	Japan	2016	17	75 (52–81)	6/9	44 (8–96)		92 (21–209)	37 (9–93)	iv
Robinson et al. [13]	USA	2015	15	62 (44–83)	6/9	24 (3–68)		52 (21–83)	16 (1–76)	iv
Brieau et al. [14]	France	2015	2	70/69	2/0	26/60	alive/alive	68/132	36/60	iv
Yamashita et al. [3]	Japan	2015	2	NA	NA	21/36	alive/alive	65/75	44/39	iv
Downs-Canner et al[4]	USA	2015	8	NA	NA	NA		67.5(NA)	NA	iv
Nakada et al. [15]	Japan	2014	1	68	m	16	alive	22	4	iv
Kitasato et al. [16]	Japan	2012	4	61 (59–72)	2/2	52.5 (25–132)		69.5 (64–167)	21 (4–41)	iv
Arnaoutakis et al. [2]	USA	2011	9	69.4 (5.5) (mean, SD)	3/6	29 (18–47)		51 (39–53)	18.6 (5.6–29.2)	iv

Moon et al. [17]	USA	2011	1	56	m	24	alive	39	11	iv
Groot, et al.[18]	USA	2019	19	65 (60 – 74)	NA	24.3(9.6-83.0)		68.9 (45.5 – 92.3)	35.0 (21.1 – 48.9)	iv
Okui, et al. [19]	Japan	2017	6	64(53-74)	3/3	26.0(19.7-64.0)		85.9(65.4-114.6)	38.3(31.1-47.7)	iv
IImer M, et al.[20]	Germany	2019	11	66 (49 – 75)	6/5	17		37.7	26	iv

i: synchronous metastasis: no surgical treatment

ii: metachronous metastasis: no surgery for primary tumor and metastasis

iii: metachronous metastasis: resection of the primary tumor, no surgery for metastasis

iv: metachronous metastasis: resection of the primary tumor and metastasis

survival 1: survival after the initial diagnosis/ surgery

survival 2: survival after the diagnosis of lung metastasis/ resection of metastasis

NA: not available

Table S3: Extracted information of 79 patients from the reported studies.

Age	Gender	Differentiation(G1/G2/G3/G4/unknown)	Stage (I/II/III/IV/ unknown)	Number of lesions	Interval	Status	Survival 1	Survival 2
70	m	0/1/0/0/0	1/0/0/0/0	3	56	alive	96	40
83	NA	0/0/0/0/1	0/1//0/0/0	1	15	dead	25	10
78	NA	0/0/0/0/1	0/0/1/0/0	3	8	alive	43	35
64	NA	0/0/0/0/1	0/1//0/0/0	1	42	alive	75	33
78	NA	0/0/0/0/1	0/1//0/0/0	1	50	alive	83	33
72	NA	0/0/0/0/1	0/1//0/0/0	2	39	alive	72	33
75	NA	0/0/0/0/1	0/0/0/1/0	1	49	dead	71	24
77	NA	0/0/0/0/1	0/0/1/0/0	2	11	dead	23	12
77	NA	0/0/0/0/1	0/1/0/0/0	1	74	dead	121	47
63	NA	0/0/0/0/1	0/1/0/0/0	1	24	alive	90	66
70	NA	0/0/0/0/1	0/0/1/0/0	3	30	dead	61	31
80	NA	0/0/0/0/1	0/0/1/0/0	1	34	alive	62	28
74	NA	0/0/0/0/1	1/0/0/0/0	1	22	alive	28	6
NA	f	0/0/0/0/1	0/1/0/0/0	1	34	alive	45	11
NA	f	0/0/0/0/1	0/1/0/0/0	1	52	alive	65	13

66	f	0/0/0/0/1	0/0/1/0/0	4	96	dead	209	113
76	f	0/0/0/0/1	0/0/1/0/0	1	61	dead	120	60
79	f	0/0/0/0/1	1/0/0/0/0	1	8	dead	21	9
75	m	0/0/0/0/1	0/1/0/0/0	1	53	dead	66	15
81	m	0/0/0/0/1	0/0/1/0/0	1	51	dead	66	15
79	f	0/0/0/0/1	0/0/1/0/0	1	72	dead	90	18
69	f	0/0/0/0/1	0/1/0/0/0	2	26	dead	58	32
77	f	0/0/0/0/1	0/0/1/0/0	8	78	dead	92	14
68	f	0/0/0/0/1	0/1/0/0/0	1	37	dead	130	93
78	f	0/0/0/0/1	0/1/0/0/0	2	31	dead	114	83
68	m	0/0/0/0/1	0/1/0/0/0	1	96	dead	112	16
52	m	0/0/0/0/1	0/1/0/0/0	2	54	dead	68	12
79	f	0/0/0/0/1	0/0/1/0/0	3	20	dead	65	42
78	f	0/0/0/0/1	1/0/0/0/0	1	39	alive	97	58
66	m	0/0/0/0/1	1/0/0/0/0	1	31	alive	106	75
71	f	0/0/0/0/1	1/0/0/0/0	1	60	alive	97	37
60	m	0/0/0/0/1	0/1/0/0/0	1	36	alive	74	38
76	m	0/0/0//0/1	0/1/0/0/0	NA	30	alive	78	48
58	f	0/0/0/0/1	0/1/0/0/0	NA	22	dead	46	24

44	f	0/0/0/0/1	0/1/0/0/0	NA	24	alive	57	33
59	m	0/0/0/0/1	0/1/0/0/0	NA	18	alive	45	27
56	m	0/0/0/0/1	0/1/0/0/0	NA	29	alive	45	16
65	f	0/0/0/0/1	0/1/0/0/0	NA	24	dead	57	33
56	f	0/0/0/0/1	0/0/0/0/1	NA	68	dead	83	15
73	f	0/0/0/0/1	0/1/0/0/0	NA	8	dead	36	28
62	m	0/0/0/0/1	0/1/0/0/0	NA	52	dead	68	16
62	f	0/0/0/0/1	0/1/0/0/0	NA	3	alive	79	76
76	m	0/0/0/0/1	1/0/0/0/0	NA	43	dead	46	3
42	m	0/0/0/0/1	1/0/0/0/0	NA	68	alive	72	4
83	f	0/0/0/0/1	0/1/0/0/0	NA	22	alive	25	4
64	f	0/0/0/0/1	0/1/0/0/0	NA	48	alive	52	3
61	f	0/0/0/0/1	0/1/0/0/0	NA	20	alive	1	1
70	m	1/0/0/0/0	0/1/0/0/0	1	26	alive	68	36
69	m	1/0/0/0/0	0/1/0/0/0	1	60	alive	132	60
NA	NA	0/0/0/0/1	0/0/0/0/1	1	21	alive	65	44
NA	NA	0/0/0/0/1	0/0/0/0/1	1	36	alive	75	39
68	m	1/0/0/0/0	0/1/0/0/0	1	16	alive	22	4
NA	NA	0/0/0/0/1	0/0/0/0/1	NA	63	alive	63.7	0.7

NA	NA	0/0/0/0/1	0/0/0/0/1	NA	36	alive	55	19
NA	NA	0/0/0/0/1	0/0/0/0/1	NA	52	alive	100	48
NA	NA	0/0/0/0/1	0/0/0/0/1	NA	14	alive	18	4
NA	NA	0/0/0/0/1	0/0/0/0/1	NA	86	alive	103	17
NA	NA	0/0/0/0/1	0/0/0/0/1	NA	55	alive	84	29
NA	NA	0/0/0/0/1	0/0/0/0/1	NA	22	alive	52	30
56	M	0/0/0/0/1	0/1/0/0/0	1	24	alive	39	11
NA	NA	0/0/0/0/1	0/0/0/0/1	NA	63	alive	63.7	0.7
NA	NA	0/0/0/0/1	0/0/0/0/1	NA	36	alive	55	19
NA	NA	0/0/0/0/1	0/0/0/0/1	NA	52	alive	100	48
NA	NA	0/0/0/0/1	0/0/0/0/1	NA	14	alive	18	4
NA	NA	0/0/0/0/1	0/0/0/0/1	NA	86	alive	103	17
NA	NA	0/0/0/0/1	0/0/0/0/1	NA	55	alive	84	29
NA	NA	0/0/0/0/1	0/0/0/0/1	NA	22	alive	52	30
NA	NA	0/0/0/0/1	0/0/0/0/1	1	13.3	dead	39.6	17.7
NA	NA	0/0/0/0/1	0/0/0/0/1	1	48.9	dead	134.1	83.9
NA	NA	0/0/0/0/1	0/0/0/0/1	1	40.3	alive	103.0	38.4
NA	NA	0/0/0/0/1	0/0/0/0/1	5	23	dead	58.0	24.9

NA	NA	0/0/0/0/1	0/0/0/0/1	1	61．8	alive	136.1	71.6
NA	NA	0/0/0/0/1	0/0/0/0/1	1	11．4	dead	33.8	18.5
NA	NA	0/0/0/0/1	0/0/0/0/1	2	83．0	alive	104.7	17.1
NA	NA	0/0/0/0/1	0/0/0/0/1	1	35．8	alive	75.0	37.5
m	64	0/0/0/0/1	1/0/0/0/0	1	64	alive	114.6	47.7
m	65	0/0/0/0/1	0/1/0/0/0/	2	25．1	alive	91.7	46.9
f	63	0/0/0/0/1	0/1/0/0/0	1	19．7	alive	77.6	36.2
m	65	0/0/0/0/1	0/0/0/0/1	1	54．1	alive	85.9	31.1

Survival 1: survival after initial tumor resection

Survival 2: survival after resection of metastasis

NA: not available
